# Association between SNPs and Loss of Methylation Site on the CpG island of the Promoter Region of the Smoothened Gene, Potential Molecular Markers for Susceptibility to the Development of Basal Cell Carcinoma in the Brazilian Population

**DOI:** 10.31557/APJCP.2020.21.1.25

**Published:** 2020

**Authors:** Augusto Monteiro de Souza, Otávio Sérgio Lopes, Andressa de Lima Liberato, Paulo Junior Ribeiro de Oliveira, Sylvia Satomi Takeno Herrero, Agnaldo Luiz do Nascimento, Carlos Alberto Longui, Ivan Rodrigues de Carvalho Filho, Leonardo Ferreira Soares, Renally Barbosa da Silva, Rommel Rodriguez Burbano, Plínio Delatorre, Eleonidas Moura Lima

**Affiliations:** 1 *Laboratory of Structural Molecular Biology and Oncogenetics - LBMEO, *; 2 *Postgraduate Program in Cellular and Molecular Biology, Federal University of Paraiba, *; 3 *Department of Dermatology, Dermatological Clinic Santa Catarina, *; 4 *Depathology of Pathology, Laboratory of Medical Analysis - UNILAB, *; 9 *Molecular Biology Department; Federal University of Paraiba; João Pessoa - PB, *; 4 *Postgraduate Program in Health Sciences; Santa Casa School of Medical Sciences of São Paulo; Sao Paulo – SP, *; 6 *Pharmacy Department; State University of Paraíba; Campina Grande – PB, *; 7 *Postgraduate Program in Natural Sciences and Biotechnology, Federal University of Campina Grande - UFCG, Campus de Cuité – PB,*; 8 *Institute of Biological Sciences; Federal University of Pará; Belém - PA - Brazil Brazil. *

**Keywords:** Basal Cell Carcinoma, CpG-SNPs, via Hedgehog, molecular markers e DSAPS

## Abstract

**Objective:**

Perform genotyping of SNPs in the promoter region of the SMO gene in BCC samples from patients from northeastern Brazil, and to determine if there is an association of these SNPs of the gene in question with the susceptibility to the development of the BCC.

**Methods:**

100 samples of paraffined tissue from patients with histopathological diagnosis of BCC and 100 control samples were analyzed for each polymorphism by a newly developed genotyping method, the Dideoxy Single Allele Specific – PCR. The software Bioestat - version 5.3 and Haploview 4.2 were used for the statistical analysis. For all tests a P-value <0.05 was considered significant.

**Results:**

The SNP rs538312246 is the Hardy-Weinberg equilibrium, therefore, it did not present significant association with the BCC (X² =2.343 and P<0.158). However, the CpG-SNPs rs375350898 and rs75827493 were significantly associated to the BCC in the analyzed samples (X^2^ = 27,740/21,500 and P <0001), the SNP rs75827493 showed a significant association with the BCC of the nodular subtype (P <0.0069). Therefore, our results suggest that SNPs rs375350898 and rs75827493 are potential molecular markers for susceptibility to BCC.

**Conclusion:**

The ability to detect SNP in a population, especially in promoter regions, has profoundly changed human genetic studies. This study allowed the understanding of the relationship between the presence of SNPs in CpG islands of the promoter region of the SMO gene can modify the methylation pattern and provide susceptibility to BCC in the population.

## Introduction

Basal cell carcinoma (BCC) is a cutaneous neoplasm that originates from immature pluripotent epithelial basal cells that have lost their capacity for differentiation, normal keratinization and cutaneous appendages development (Narala and Cohen, 2016). BCC is one of the most frequent types of malignant skin tumors, with slow growing prominent lesions and invasive potential, but metastasis are rare (Kosiniak-kamysz et al., 2012).

Studies have shown that the incidence of skin cancer varies greatly between different countries and different ethnicities, suggesting that genetic factors play important roles in their development (Chen et al., 2014). The BCC is a complex disease with influence from the interaction between constitutional predisposition (genotypic, phenotypic and environmental characteristics) and subsequent exposure to environmental risk factors for its development (Verkouteren et al., 2017).

The Hedgehog pathway is an evolutionarily conserved signaling mechanism that allows gradual responses of cells and tissues to control fundamental biological processes, such as target cell specification, tissue standardization, proliferation regulation, and maintenance of tissue homeostasis. The activation of the pathway is also essential for the proper development of the skin and its appendages, but increased activation can lead to hyperproliferation of the epidermis, defects in differentiation and cancer (Atwood et al., 2015).

BCCs are characterized by aberrant upregulation of the Hedgehog pathway, typically through loss of the Patched 1 receptor or activation of the Smoothened G protein coupled receptor (SMO) (Amakye et al., 2013), resulting in the deregulation of GLI transcription factors, processes involved in cell growth and proliferation. Recently, mutations in the Hedgehog pathway have been identified in genome-wide sequencing studies across a wide range of cancers. Interestingly, mutations in the gene were detected in other types of cancer (Otsuka et al., 2015). In recent years, the Hedgehog signaling pathway has been shown to be an essential element in tumor initiation or in more advanced tumor evolution. Studies show that this pathway is deregulated in more than 30% of human cancers, including basal cell carcinoma (BCC), medulloblastoma (MB), melanoma, breast, prostate, lung, pancreas, cervical and cervical cancer (Szkandera et al., 1999).

Single nucleotide polymorphisms (SNP) are variations on a single nucleotide generating altered sequences (alleles), where the less frequent allele has an abundance of about 1% or more (Brookes, 1999). They are the most common types of human genetic variation. Several studies have been developed with the aim of identifying sets of SNPs that serve as markers for predisposition to multifactorial diseases or for mapping of linkage disequilibrium in all genomes (Takahashi et al., 2008). Pesz et al., (2014) reports that several case studies have investigated the influence of various SNPs on the development of BCC. 

Several studies have associated a number of SNPs in promoters with the occurrence of methylation-induced silencing in large series of cancers (Fennell et al., 2018). Genetic changes such as SNPs in genes involved in the Hedgehog pathway, may result in individuals susceptible to the development of BCC. From this perspective, we aimed to study polymorphisms in CpG islands (in the promoter region of the SMO genes) of the BCC, to determine if there is an association of these SNPs with the susceptibility to the development of the BCC.

## Materials and Methods

*Samples*

This is a retrospective study which included up to five-year-old samples from a database supplied by Laboratório UNILAB/João Pessoa - Brazil- sample bank PB - and LABMEO/UFPB. A hundred samples of tissue embedded in paraffin from patients with histopathological diagnosis of BCC were analyzed, totaling 200 alleles for each studied SNP. Additional 100 control samples with normal tissue from cancer free patients were evaluated, obtained from the Laboratory of Molecular Structural and Oncogenetic Biology - Sample bank of LABMEO / UFPB. The present study is part of the thematic project approved by the Ethics Committee of the University Hospital Lauro Wanderley - UFPB under the code CAAE: 36.522.614.2.3001.5883.

*DNA extraction*

The samples were submitted to DNA extraction in the Laboratory of Structural Molecular Biology and Oncogenetic - LBMEO/ UFPB. The DNA sample was extracted according to the method described by Shang Rong-Shi et al. (2002), with modifications. The isolated genomic DNA was quantified with the Spectrophotometer NanoDrop™ 2000c from Thermo Fisher Scientific and stored at -20ºC.

*Dideoxy single allele-specific PCR Method*

The Dideoxy single allele-specific PCR (DSASP) method is based on chain-terminating inhibitors, dideoxynucleotide, established by the method of Sanger et.al. DSASP was previously validated by the Allele Specific PCR-ASP method as described by Lima et al., (2015). It is a method of genotyping and comprises four stages: I - selection of target SNPs, oligonucleotides (primer and complementary sequence) design and determination of the dideoxynucleotide to be incorporated; II - asymmetric PCR of target dideoxynucleotide; III - hybridization reaction between the complementary sequence and athe symmetric PCR product; IV - analysis by melting curve by qPCR, validated and developed by Lima et al. (2015).

To genotype the SNPs rs75827493, rs375350898 and rs538312246 from the SMO gene through the DSASP method, asymmetric PCR was performed for each target SNP, with complementary sequencing and dideoxynucleotide incorporation. The oligonucleotides were obtained by in silico validation (GeneRunner Software).

*Validation in silico*

The primers used for DSASP of the rs75827493, rs375350898 and rs538312246 were designed based on the Ensembl Genome Browser database, using the Gene Runner program (Table 1) to evaluate the annealing temperature, the formation of secondary structure and size of the amplified fragments.

*Asymmetric PCR conditions*

Asymmetric PCR was performed in a final volume of 25 μL containing 200 μM dNTP (dATP, dCTP, dTTP and ddGTP), 2.0 mM MgCl2, 20 ng/μL DNA, 200 pM primer and 1 U AmpliTaq Gold (Life Technologies - Carlsbad, CA). PCR conditions for amplification of single stranded DNA were as follows: a pre-denaturation for 3 min at 94°C, 80 cycles of 94°C denaturation for 20sec, annealing 60°C for 45sec and extension 72°C for 30sec with a final extension of 5 min at 72°C.

*Hybridization conditions*

The product of the PCR amplification of each sample went through the hybridization step under the following conditions: 200 pM of the complementary sequence at 4°C for 10 min.

*Melting Curve Analysis*

The melting curves were analysed to determine the Tm, performed by the Real Time PCR equipment 7,500 Fast Real-Time PCR System (Life Technologies - Carlsbad, CA) following these conditions: preheating (starting at 25ºC to 95ºC) for 1 min, folding up to 60ºC for 5 min and gradual heating (1°C/min) until a temperature of 95°C for 5 min. For melting curve analysis, SYBR^®^ Green Mix (Life Technologies - Carlsbad, CA) was used.

*Statistical Analysis*

Allele frequencies and genotypes were calculated by allele counting and the Hardy-Weinberg Equilibrium (EHW) was tested using the X^2^ test. To analyze the linkage disequilibrium between the polymorphisms, the D’ parameter was calculated using the Haploview 4.2 Software. Polymorphisms were considered in linkage disequilibrium when the D’ values were ≥ 0.75. To verify the distribution of genotypes of polymorphisms in the studied population and the distribution of age, sex, tumor location and histological type in the patient population, the Chi-Square test and the Fisher’s exact test were used through the Bioestat Software - version 5.3. For all tests a P-value <0.05 was considered significant.

## Results

Of the three CpG-SNPs studied, rs75827493 and rs375350898 were associated with the development of BCC in the analyzed samples, whereas the polymorphism rs538312246 of the SMO gene was in the Hardy-Weinberg equilibrium (Table 2).

The genotype frequencies of the SMO gene rs75827493 polymorphism were as follows: 31% (n = 31) G / G, 69% (n = 69) T / G and 0% (n = 0) T / T. The frequency of the polymorphic allele (T) corresponds to 34.5% within the studied samples; the observed / expected genotypic frequency ratio indicates that it is in Hardy-Weinberg imbalance with X² = 27,740 and P-value <0.0001. The Melting temperature (Tm) observed for the T allele varied between 79°C and 81°C, while the G allele had a Tm between 86°C and 88°C. For the TT allele this temperature represents the smallest double-stranded DNA fragment obtained by the DSASP technique. The larger fragment of the amplified DNA represents the GG genotype.

The genotype frequencies of the polymorphism rs375350898 of the SMO gene were as follows: 11% (n = 11) T / T, 73% (n = 73) T / G and 16% (n = 16) G / G. The frequency of the polymorphic allele corresponds to 47.5% in the samples studied; the observed / expected genotypic frequency ratio indicates that the SNP is in Hardy-Weinberg imbalance. X² = 21,500 and P-value <0.0001. The Tm observed for the T allele ranged from 79°C to 82°C, while the G allele showed a Tm between 89°C to 92°C.

The genotype frequencies of the SMO polymorphism rs538312246 were as follows: 04% (n = 04) A / A, 21% (n = 21) A / G and 25% (n = 25) G / G. The frequency of the polymorphic allele corresponds to 14.5% in the studied samples; the observed / expected genotypic frequency ratio indicates that the SNP is in Hardy-Weinberg equilibrium. X^2^ = 2.333 and P-value <0.1258. The Tm observed for allele A ranged from 79°C to 82°C, while the G allele showed a Tm between 89°C and 92°C.

The allelic segregation of the studied polymorphisms was also evaluated in order to verify if they were in linkage disequilibrium (DL). The analysis was performed using the Haploview software. The study showed significant linkage disequilibrium between the polymorphisms rs375350898 and rs75827493, with D ‘= 0.909, r² = 0.571. The distance between them is approximately 0.1kb. The two SNPs were grouped into one block. Groups of less than 1% are not shown in the analysis. The frequencies found were: GGG 44.9%, TTG 29.6%, TGG 9.5%, TTA 6.5%, GGA 6.2%, GTG 1.4% and TGA 1.3%. The red color indicates strong linkage disequilibrium (D ‘= 100, non-random segregation of alleles), while white demonstrates those with weak (D’ <100) or absent (D’= 0, independent segregation of alleles) imbalance.

Analysis of the association of the genotypes found for CpG-SNP rs75827493 (T> G) of the SMO gene with the variables of: gender, age, anatomical localization of the neoplasia and histopathological types. Our results suggest that there was statistical significance among genotypes of the histopathological types (p=0.0069) only in CpG-SNP rs75827493, but for the other variables studied, there was no statistical significance.

**Table 1:** SMO Gene Promoting Region Polimorfism List, with Corroesponding Allelles and Complimentary Sequences to be Amplified by the DSASP Method

SNP’S	Allele	Primer / Complementary Sequence
rs75827493	G/T	5’GCGCTGGGGCGAAGGTGGCTGCT3’
		5’AGCCMGCGGCCCAGCAGCCACCTTCGCCCCAGCGC3’
rs375350898	G/T	5’TGCGCCGAGGCTKGGGGAGGGACT3’
		5’GCTCCTTCKTCCAGTCCCTCCCCMAGCCTCGGCGCA3’
rs538312246	G/A	5’AGCCGGAGCTGCACTCGCACCC-3’
		5’GSCAGACGYGGGCCGGGGGTGCGAGTGCAGCTCCGGCT3’

**Table 2:** Genotypic Distribution and Allelic Frequency of SNPs in the Analyzed Gene

Samples	Gene/SNP	Genotypic distributions			X^2^	P-value
	SMO/*rs75827493*	TT	TG	GG		
BCC		0	69	31	27.740	0.0001
Controle		12	45	31		
	SMO/*rs375350898*	TT	TG	GG		
BCC		11	73	16	21.500	0.0001
Controle		22	50	28		
	SMO/*rs538312246*	AA	AG 21	GG		
BCC		04	21	75	2.343	0.1258
Controle		02	25	73		

## Discussion

DNA sequence variation analysis provides a powerful tool for understanding susceptibility to disease. The SNPs may alter the biological properties of the encoded protein and affect the levels of gene expression in an allele specific manner. Uncontrolled activation of the Hedgehog pathway leads to tumor progression in various types of cancer, including basal cell, medulloblastoma, pancreatic, colon, lung, breast, prostate and blood cancer (Amakye et al., 2013).

SNPs in coding regions are proposed as being associated with multiple diseases. However, the study of SNPs in intron regions and promoter regions are important for understanding the mechanism of genetic regulation of carcinogenesis. Calixto et al., (2017) suggest that the substitution of the base in a DNA sequence in an intronic region may alter gene regulation at the post-transcriptional level, resulting in new processing sites, altering Splicing regulatory elements and causing cancer formation.

This is the first study to report the association between polymorphisms in CpG islands in the promoter region of the SMO gene with the risk of basal cell carcinoma, it was studied that SNPs-CpG could influence DNA methylation levels provided function gain mutations in the SMO and that the protein associated with the Hedgehog pathway may allow the susceptibility to the development of BCC.

We performed the study of three SNPs in the CpG islands in the SMO gene promoter region. CpG-SNP rs75827493 and rs375350898 had polymorphic allele frequencies of 34.5% and 47.5% (respectively) and, by presenting Hardy-Weinberg disequilibrium and p<0.0001, were probably associated with the development of BCC. Haplotype analysis represent a powerful approach in the search for disease-causing alleles. In the location of the underlying mutations, we observed that there was a linkage disequilibrium between these two significantly associated polymorphisms.

Our studies corroborate the work of Kaminsky et al., (2009), which demonstrates that DNA methylation levels in specific locus are under genetic control. This has gained strength in recent years, reinforced by the results obtained in the comparison of DNA methylation patterns between monozygotic and dizygotic twins. 

Our results suggest that due to the mutability of CpG-SNPs are an important class of regulatory polymorphisms and link genetic variation to individual epigenome variability.

Recently, several studies have suggested that variations in the genetic code are associated with DNA methylation levels at CpG island sites near the promoter regions. Dayeh et al., (2013) observed that 19 of the 40 (48%) SNPs studied were associated with type 2 diabetes, and these SNPs could introduce or remove a CpG site, they generated successful DNA methylation data for 16 of these 19 CpG-SNPs. They studied several candidate genes for type 2 diabetes and all analyzed CpG-SNPs were associated with differential DNA methylation of the CpG-SNP site in the promoter region.

The presence of some SNPs located in CpG dinucleotides can generate or eliminate a CpG island site, called CpG-SNPs. CpG-SNPs are considered to adjust the interaction of genetic (SNP) and epigenetic (DNA methylation), which affect the mechanisms of gene expression enabling the activation or inactivation of the gene (Chen et al., 2018).

It is believed that these CpG SNPs in gene promoter regions are associated with multiple diseases, including type 2 diabetes (Dayeh et al., 2013), coronary disease (Xu et al., 2015; Chen et al., 2016), breast cancer (Harlid et al., 2011; Chen et al., 2018) and epithelial ovarian cancer (Koestler et al., 2014). Our results will allow us to put together a new line of research within the BCC study. 

In general, Koestler et al., (2014) have contributed to the growing collection of integrative genomics studies, exploring the relationship between genetics and epigenetics and the risk of epithelial ovarian cancer. The authors identified 17 CpG-SNPs that represent potentials mediated by methylation between the genotype and the risk of epithelial ovarian cancer. These findings provide additional information about the etiology of the disease and may serve as new biomarkers for cancer susceptibility.

The SNP rs538312246 showed a frequency of the polymorphic allele corresponding to 14.5% and the observed / expected genotypic frequency ratio indicates that the SNP is in Hardy-Weinberg equilibrium with P-value <0.1258.

Our results also found significant association of SNP rs75827493 among histopathological genotypes (p = 0.0069). For 21. Chen et al., (2014) the development of carcinogenesis of BCC presents a higher incidence of nodular pattern in the population.

The ability to detect SNP in a population, especially in promoter regions, has profoundly changed human genetic studies. One of the major concerns of polymorphism studies has been to analyze markers for mapping and identifying genes that cause cancer. This study allowed the understanding of the relationship between the presence of SNPs in CpG islands of the promoter region of the SMO gene can modify the methylation pattern and provide susceptibility to BCC in the population.
